# Greening of Daycare Yards with Biodiverse Materials Affords Well-Being, Play and Environmental Relationships

**DOI:** 10.3390/ijerph16162948

**Published:** 2019-08-16

**Authors:** Riikka Puhakka, Outi Rantala, Marja I. Roslund, Juho Rajaniemi, Olli H. Laitinen, Aki Sinkkonen, the ADELE Research Group

**Affiliations:** 1 Ecosystems and Environment Research Programme, Faculty of Biological and Environmental Sciences, University of Helsinki, Niemenkatu 73, FIN-15140 Lahti, Finland; 2 Multidimensional Tourism Institute, University of Lapland, Viirinkankaantie 1, FIN-96300 Rovaniemi, Finland; 3 Faculty of Built Environment, Tampere University, Korkeakoulunkatu 5, FIN-33014 Tampere, Finland; 4 Faculty of Medicine and Health Technology, Tampere University, Arvo Ylpön katu 34, FIN-33520 Tampere, Finland

**Keywords:** affordance, children, well-being, physical activity, environmental relationship, green space, biodiversity

## Abstract

Nature contacts are recognized as positively contributing to humans’ health and well-being. Although there have been projects to green daycare or schoolyards, yard greening and microbial biodiversity have never been studied simultaneously. We asked whether simultaneously increasing biodiversity exposure and greening urban daycare yards affects 3–5 years-old children’s physical activity and play, their environmental relationships, and their perceived well-being. For transforming six daycare yards in Finland, we used a forest floor with high biodiversity, sod, peat blocks, and planters for vegetable and flower growing. We used qualitative interview and survey-based data collected from the daycare personnel and parents to analyze how green yards encourage children’s engagement with their everyday life-worlds. We identified the functional possibilities provided by the yards and the dynamic aspects related to the greening. Green, biodiverse yards were considered safe, and inspired children’s play, diversified their activities, and increased physical activity. The greenery offered embodied experiences of nature and provided the children with multi-sensory exploration and diverse learning situations. The dynamic and emotional ways of engaging with the natural environment increased their well-being. The activities related to caring for the yards and exploring them promoted the development of environmental relationships. The results can be used for designing health-enhancing yards

## 1. Introduction

In urbanized societies, children’s opportunities to connect with nature in everyday life have diminished as the number of natural areas has decreased, and the level of children’s independent mobility has declined [1,2]. While acknowledging the inextricable links between human health and the health of natural systems [3], contact with nature is recognized as positively contributing to children’s and adults’ psychological, physiological and social well-being and health [4,5,6]. For instance, interacting with nature increases self-esteem and mood and has positive effects on emotions and behavior. Natural areas are restorative and contribute to attentional recovery and the reduction of mental fatigue [7,8,9]. Exposure to nature has positive effects on both adults’ and children’s concentration, academic performance, and the ability to perform mentally challenging tasks [10,11,12]. In terms of physiological benefits, studies conducted among adults and children show that contacts with nature alleviate the negative effects of various stressors in urban environments [12,13], and natural settings activate people to move [14]. Physical activity benefits not only mental and physical health, but also general child development, because movement stimulates brain development [15].

Furthermore, exposure to greenness in early childhood is associated with benefits to the immune system and health [16]. Contact with diverse environmental microbiota affects the human commensal microbiota and drives effective functioning of the immune system that persists into adulthood. Decreased biodiversity, due to high hygiene levels and modern urban lifestyles, may result in imbalanced human microbiota and a higher risk of immune-mediated diseases, such as allergies, asthma and Type 1 diabetes [17,18]. Recent evidence also suggests that gut microbiota might play a potential role in mental health through neurotransmission pathways [19].

In urbanized societies, school or daycare grounds can be one of the primary opportunities for children’s contact with nature and highly important for their social, emotional, physical and cognitive development [20,21]. The umbrella term ‘greening’ is used to include a range of changes, including naturalization, habitat restoration, tree planting, food gardening, and similar efforts to bring nature back to urban space [22].

The benefits of green school or daycare yards include increased play opportunities and more creative, unorganized ‘free’ play [23,24,25,26], which is more inclusive for children of different sexes, ages and competences than play in barren settings [22,27,28,29]. Green yards have been found to enhance relationships with the natural world and heighten environmental concern [24,30]. Green yards have enhanced children’s attentiveness [31,32,33], increased learning opportunities, and improved children’s student performance [34,35]. Furthermore, green yards have promoted children’s physical activity [29,36,37] and motor development [38], and enhanced physical and mental health [31,39]. Green yards have been associated with reduced physiological stress levels, higher self-reported psychological well-being [40] and restorativeness [41], as well as with healthier body shape and longer night sleep [42]. Despite intensive research, yard greening and microbial biodiversity have never been studied simultaneously although having hands in the soil and playing with biodiverse materials presumably increases children’s contact with diverse environmental microbiota, diversifies their skin microbiota and may benefit health [43,44,45].

In this study, we examined whether simultaneously increasing biodiversity exposure and greening daycare yards is perceived to affect 3–5 years-old children’s physical activity and play, their environmental relationships, and their well-being in the urban environment in Finland. We applied the concept of ‘affordance’ [46] to analyze how green yards encourage children’s engagement with their everyday life-worlds and enhance well-being in daycare centers. We were also interested in how direct engagement with green yards may provide experiences that support the development of a dynamic relationship between children and their surroundings.

## 2. Materials and Methods

### 2.1. Theoretical Framework

James J. Gibson’s [46] concept of affordance has been applied in diverse ways in studies of children’s outdoor play in natural environments [27,47,48,49,50]. Basically, the concept points to the material possibilities and restrictions emerging from the environment, which are perceived as functionally meaningful units—for instance, as surfaces to be climbed or objects to be lifted [46,48]. In the ontology and epistemology of the concept of affordance, the immediate interaction between the environment and the perceiver is central: Affordances exist in relation to the perceiver, but cannot be constructed subjectively by the perceiver [46]. Furthermore, theoretical tension has existed between those who see affordances as available resources and those who see them as relational [51]. Recent studies on children’s outdoor play have highlighted that it is important not to concentrate more on the characteristics of an environment than on those of children [47].

In the relational tradition of applying the concept of affordance, children are seen as active and influential participants in their own life-worlds, and the relationships between children and their surroundings are formed in the processes of everyday living and curious play [47,52]. This approach resembles the phenomenological approaches to play, which do not see the surrounding environment as a separate object but as something children take with them into their own experiences [53]. Relational and phenomenological approaches see the richness of the concept of affordance as relating to the role of pre-reflexive bodily sensing and the development of skills [54], which are highlighted in children’s relationships with their life-worlds [47,52]. According to Ingold [55] (p. 168), “Gibson assumed that the world which the perceiver moves around in and explores is relatively fixed and permanent, somehow prepared with all its affordances ready and waiting to be taken up”. From the relational and phenomenological standpoints, in contrast, the world emerges with its properties alongside the emergence of the perceiver in person, against the background of involved activity [55] (p. 168). Children investigate their surrounding nature by intertwining with the environment and creating simultaneously novel affordances that invite them to curiosity and wonder [47].

In this paper, we apply a relational approach to affordances in order to pay attention to how the greening of daycare yards with biodiverse materials is perceived to affect children’s physical activity and play, their well-being and environmental relationships—and to understand better teachers’, child nurses’ and children’s role in this process.

### 2.2. Study Setting

**Figure 1:**
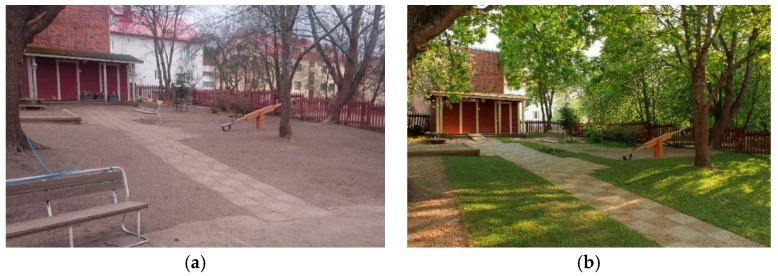
(**a**) Before the intervention, the daycare yards were mostly covered by mineral soil materials; (**b**) After the intervention, the daycare yards were covered by forest floor mat and sod. Photos: Aki Sinkkonen (**a**) and Mira Grönroos (**b**).

We analyzed how green yards with diverse vegetation and high microbial load encourages children’ engagement with their everyday life-worlds and enhance well-being in daycare centers that are located in urban areas in southern Finland. The yards were transformed by replacing areas covered by mineral soil materials, such as gravel, with forest floor (around 100 m^2^ per yard, provider Piiraisen Kuntta Oy, Kajaani, Finland), sod (around 200 m^2^ per yard, provider Mesiän Siirtonurmi, Hämeenkoski, Finland), peat blocks (provider Kekkilä Oy, Vantaa, Finland), and planters for vegetable and flower growing (Figure 1). Due to the transferability of the materials, it took only one night to green the yards. The materials were easy to find and relatively inexpensive. The personnel introduced the green materials into the children’s various activities with the help of tips from an environmental educator (including a written description of 13 nature-based activities, several crafting ideas, and links for further information). The children also used the yard freely when they spent time outdoors every day (0.5–2 h in the morning and in the afternoon).

The forest floor mat was transplanted as vegetated 10 m^2^ pieces of soil surface (Ø 10 cm). The vegetation consisted mainly of edible dwarf shrubs, such as crowberry (*Empetrum nigrum*), blueberry (*Vaccinium myrtillus*), lingonberry (*Vaccinium uliginosum*), and heather (*Calluna vulgaris*), an abundant moss layer (major taxa comprising *Pleurozium schreberi*, *Hylocomium splendens*, *Dicranum* sp., *Shpangnum* sp.), and wooden plant parts, such as Scotch pine (*Pinus sylvestris*) cones and sticks. These species were naturally growing on the forest floor, and they belong to the most widespread species in boreal vegetation zone. The sod consisted of fescues (*Festuca* sp.) and meadow grasses (*Poa* sp.).

**Figure 2:**
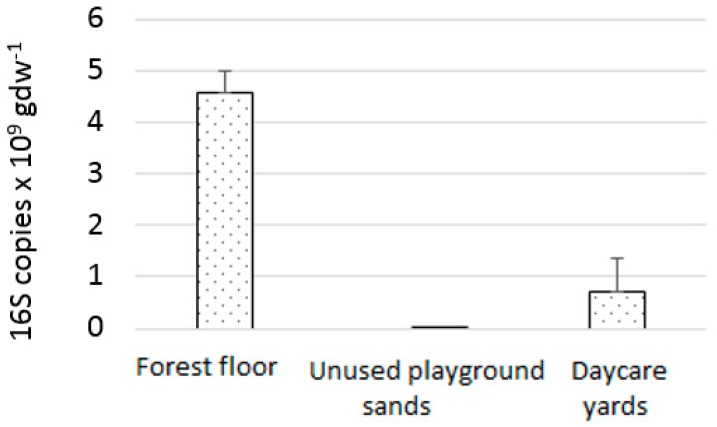
Bacterial numbers in the forest floor, unused playground sands, and samples from daycare yards taken before transforming the yards. 16S copies = copies of bacterial 16 S rRNA sequences per 0.250 g sample; gdw = gram dry weight.

**Figure 3:**
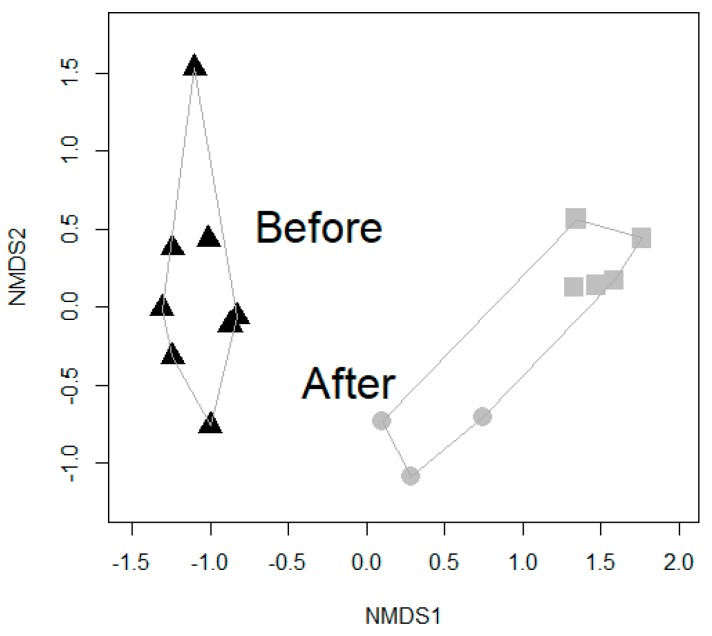
Non-metric multidimensional scaling (NMDS) at the OTU level showed that bacterial communities differed after the daycare yards were transformed with green materials. Triangle = mineral soil, Circle = sod, and Square = forest floor. OTU = operational taxonomic unit with a 97% identity, an operational definition used to classify groups of closely related individuals. OTU is often used as a synonym of bacterial species.

The bacterial load of the forest floor was a magnitude higher than that of the mineral soil materials (Figure 2; molecular biological analytical methods in [56,57,58]). The composition of the bacterial community of the transformed and non-transformed yards was different (Figure 3; Table 1; statistical analyses, i.e., non-metric multidimensional scaling, PERMANOVA, and *t*-tests described in [44,59,60]). Importantly, the abundance of phyla Proteobacteria and Bacteroidetes in the modified and non-modified yards was different; changes in the abundance of these taxa have previously been associated with immune system modulation or disorders [18,61,62].

The study was conducted in three big cities (with 100,000–300,000 inhabitants) located in the most densely populated and urbanized area of Finland. In May 2016, we added greenery to four daycare yards in Lahti and Tampere, and in May 2017 to two daycare yards in Espoo. These yards were located in the city center or suburbs characterized by apartment buildings and high population density. One month after greening the yards, the participating 3–5 years-old children gave blood and/or microbial samples for analysis of blood leukocytes and skin and stool bacterial communities (data not included in this article). The guardians of all participants gave their informed consent for inclusion before the study. The study was conducted in accordance with the Declaration of Helsinki, and the protocol was approved by the Ethics Committee of the Pirkanmaa Hospital District, Finland (R16040).

### 2.3. Data

Data collected in six daycare centers included surveys completed by personnel (teachers and child nurses) and children’s parents, and interviews of personnel (Table 2). One month after greening the yards in 2016 and 2017, 13 employees from the different daycare groups (including all participating groups) completed a survey. It included both structured and open-ended questions related to the children’s activities in the modified yards, their enthusiasm and contact with greenery, and the changes that took place in the daycare center (e.g., ‘Describe your guided activities with the green materials’, ‘How excited the children in your group were about these activities?’, ‘Describe children’s excitement in more detail (e.g., which has interested them and which has not?)’, ‘Describe children’s free play and activities with the green materials (e.g., what different-aged children have done?)’, ‘Did the green materials change children’s play in the daycare center?’). All questions concerned the whole daycare group (12–24 children), not only the children who took part in the collection of blood and/or microbial samples.

Altogether 49 (of 60) parents of the children who participated in the data collection completed a survey that included open-ended questions related to the green yards (i.e., ‘Has your child told about the green materials at home? Has the child told about play or guided activities related to the green materials? What has s/he told, and how has s/he reacted to the green materials?’ and ‘Other feedback and ideas to develop green yards’).

Furthermore, twelve daycare employees from eight groups participated in individual or group interviews, which lasted from ten to thirty minutes. In thematic interviews, the teachers and child nurses were asked to describe the possible changes that took place after greening the yards—in children’s play and other activities in the yard (quantitative and qualitative changes), in children’s and employees’ interest in and knowledge of nature, in their perceived well-being, in their attitudes towards outdoor activities, and in general practices and atmosphere in the daycare center. The interviews were tape-recorded and transcribed verbatim.

### 2.4. Analysis

Survey and interview data were first analyzed using qualitative content analysis to identify different functional possibilities afforded by the green yards. We understood affordances as resources that are available to the perceivers [50]. Thus, we classified the affordances that the children perceived under six themes that were emerging from the data through analysis and coding. Second, we examined how the functional possibilities supported the development of a dynamic relationship between the children and their surroundings. At this phase, we pointed out different dynamic aspects originating from the sketched themes of functional affordances. The dynamic aspects included embodied experiences (e.g., jumping, lying on the ground), use of imagination (diverse imaginary plays), role of sensuality (e.g., odours, fresh air, touching the ground), diverse learning situations (pedagogical targets and information about nature), emotional involvement (daring, managing situations, excitement, caring, and collectivity), exploration (e.g., examining bugs and plants), and involvement (practical involvement, being dirty and wet).

Third, based on the six themes we summarized the dynamic aspects under three perspectives: (1) Changes in the embodied experiences related to the yards; (2) involvement enhanced by the greening; and (3) exploration supported by the greening. These aspects enabled us to analyze the children’s contact with nature as a relational interaction, children and nature as interacting agents, and the human-nature relationship as a dynamic and emotional relation rather than an object-subject relation [53]. Furthermore, we were especially interested in observing ‘nature happiness’—how dynamic and emotional ways of engaging with nature, or more precisely natural elements in this study, impacted on the children’s well-being [53].

## 3. Results

We first briefly introduce the functional possibilities provided by the green, biodiverse daycare yards. Then we expand our analysis to the dynamic aspects related to the greening by introducing in detail the three summarized perspectives: Embodied experiences, involvement and exploration. These perspectives are illustrated by the interview quotations of the daycare personnel (teachers or child nurses).

### 3.1. Functional Affordances

During the one-month study period, a third of the participating groups organized guided, teacher-led activities in the green yards on 4–5 days a week while a few only on 0–2 days a week. Moreover, all participating groups spent free time in the yard twice a day just as they normally do. Almost all daycare employees responded that the children were very excited or excited about the green yards and spent a lot of time in contact with the forest floor mat, sod, peat blocks, and planters. In general, the children were motivated to go outdoors, had spontaneous contact with the green infrastructure, and easily found pleasant activities in the yard. Hence, green materials enabled various functional affordances. We categorized the possibilities mentioned in the surveys or interviews under six themes: Physical activity, multi-sensory experiences, diverse play, art and crafts, nature exploration, and pre-academic skills (Table 3).

### 3.2. Embodied Experiences

Based on the data, the forest floor mat, sod, peat blocks, and planters afforded embodied experiences of green space for children. To begin with, the natural materials increased and diversified the children’s physical activity in the daycare yards. The sod and forest floor mat enabled activities, such as rolling, creeping, crawling, doing somersaults and cartwheels, or doing other physical movements, which were not favored activities in the former yard covered mainly by gravel and asphalt (Table 3). The peat blocks afforded jumping down or over, walking on, and carrying or throwing them. One teacher believed that the forest floor mat benefitted small children’s motor development because they had to raise their legs high when they walked on it. Furthermore, when the gravel under the climbing frames or swings was replaced with vegetation, some children were encouraged to climb or swing more. This illustrates the dynamic relationship between the perceiver of the affordances and the perceived environment: How the world emerges alongside the emergence of the perceiver in person [55] (p. 168).

“*Our bigger kids started on the sod, they just ran and rolled on it. It was just so lovely when they ran up and rolled down it and just lay splayed out on the slope. There used to be such terrible, dry sand there and it was dusty, the so-called lawn area. Now it’s inviting, and we’ve had blankets and books on the sod, and they’ve kind of had ‘excursions’ there. And of course, all games of tag and other games that the children have invented.*”

(Daycare center 5)

The greenery also increased opportunities for resting and relaxing in the yard—lying or sitting on the soft ground and looking at the sky or listening to different sounds. The green yard enabled social activities organized by daycare employees, such as reading stories or having a picnic. Children engaged in sensory-based play, especially in teacher-led activities. They were in contact with natural materials not only through the sense of touch, but also through smelling them and tasting grown vegetables or forest plants, such as blueberries. Moveable, loose materials, such as sticks and cones, were used to make sounds. Some teachers organized sensory exercises for the children, such as searching for different shades of green in the yard. Furthermore, the greenery afforded fresh air; when areas covered with mineral soil materials were replaced with the forest floor mat and sod, most employees felt that the amount of dust in the yard decreased.

Overall, the employees and parents felt that the modified yards were pleasant as they looked and smelled good. The greening of the yards had positive impacts on both the children’s and adults’ mood, energy, and motivation in the daycare centers. Some daycare employees noticed that when the children spent active, inspiring time outdoors, they had a good appetite at lunchtime, and slept more deeply during their naps. This was considered to further benefit the children’s well-being and learning ability.

“*Now it’s become very difficult to finish playing. They would rather continue, and those who need to take a nap, they’ve had a nice, long time outdoors and nice games so they fall asleep more easily, and it affects their energy in the afternoon. Some children have very long days here. They come in the morning and stay until five o’clock; they seem to be somehow energetic and lively in the yard. This is new for us. The contrast to the previous yard is so great that the effects can be seen here very quickly.*”

(Daycare center 6)

The embodied engagement with the forest floor mat, sod, peat blocks, and planters afforded children to dare to test their skills and capacities in new forms. The greening of the yards also enabled them to engage with the “natural” rhythms originating from their own bodies—for instance, tiredness, managing strengths and hungriness. The dynamic relationship that develops through this engagement can help in perceiving one’s own body in new ways through both practical and emotional involvement, and increase self-esteem, for instance.

### 3.3. Involvement

The green materials inspired the children’s play and enabled them to use their imagination in the yards. The children used the forest floor mat and sod as a playground for toys, but their play without toys also increased as they spontaneously engaged more in creative play and imaginative role-playing (Table 3). They built walls, forts and huts from the peat blocks, and collected loose parts, such as sticks and cones from the forest floor mat for their games (e.g., cooking food). Guided by the daycare personnel, they used plants, sticks, cones and twigs to do arts and crafts. The teachers and child nurses could feed the children’s imagination by giving them ideas for play and bringing cones and other loose parts from the forest to the yard. When the children got used to playing with natural materials, they invented new ways in which to use them, which further expanded their opportunities for creative play and enhancing well-being.

“*The children also invent themselves; when they have stimulus for their eyes, children invent it [activity] without your help. And it should be like this; some part should be like this. But you need to have stimulus. It’s not enough to have a brown yard and a climbing frame. So, it [green yard] added somehow; they definitely had good games. They pretended that they had a campfire, they got the stones and pretended that they were on a trip. And their imagination was in use there, and when children use their brains, natural tiredness arises, and it did them good, a lot of good. Then rest comes naturally, and you have a good appetite and we’re in the positive cycle. So they could use their imagination, and we encouraged them. We didn’t prohibit them, we just advised them not to rip anything.*”

(Daycare center 1)

The children also got involved with the green yards by looking after the plants and vegetation. They planted vegetables and flowers in the planters and watered and took care of them with the help of the adults; in some daycare centers, children also participated in hosing the forest floor mat and sod. The practical involvement with natural materials while caring for them—having one’s hands in soil, getting dirty and wet—enables children to get involved in feelings related to care, especially caring for their surrounding environment [63].

Furthermore, being in contact with nature and caring for it develops practical relationships in the form of becoming skilled in dressing to suit outdoor conditions. One daycare employee described how the parents seemed surprised that they had to equip their children with waterproof clothing even when it was not raining—because of the dew in the greenery. These kinds of small details related to everyday interactions with nature are highlighted in intensive involvement, but are important in enabling the development of everyday relationships with nature. One child told the nurse that the plants would grow now because they were wet. This illustrates how caring became a process of engaging and understanding through involvement in taking care of the yard.

“*They [children] go to water them [plantings], yes. Sometimes even too eager, if it’s raining. But it clearly becomes significant when the children participate in it and have done it themselves rather than if it had just been put there. […] When they have put it there themselves, it’s also important for them, and they go to see how they have grown and things like that. So it’s absolutely their project.*”

(Daycare center 6)

The children’s emotional involvement with the green yards was illustrated by their enthusiasm to take care of the plantings. Some daycare employees also thought that the green yard increased the sense of community in the daycare center, as it was something new, pleasant and inspiring that they could all enjoy, and taking care of the yard was partly their responsibility. Some employees were willing to use their own time to water the plants; they were keen to take care of their work environment and make it more pleasant for everyone.

### 3.4. Exploration

The green yards afforded the children exploration and diverse learning situations by providing opportunities for various activities and things to observe. Teachers used natural materials in environmental education, and the yards also enabled the children’s self-guided exploration and exploratory play. The children obtained information about nature when, for instance, they examined green materials with magnifying glasses, searched for bugs or worms, or identified different species on the forest floor mat with the adults’ help (Table 3). Children did this not only to learn; they also spontaneously did things like lie on the ground and observed the greenery. Planting vegetables or flowers and taking care of them enabled children to observe plant growth and raised various questions. These kinds of positive learning experiences may have positive impacts on children’s moods. When the daycare employees saw the children’s enthusiasm and the benefits of their contact with nature, they were more motivated to spend time outdoors and organize nature-based activities in the yard and trips to nearby forests.

“*Especially about the forest floor mat, I remember that our children kept asking, ‘what is it’ and ‘what’s growing there’, and explored it very carefully; they were almost lying on their stomachs there. Especially the older ones, and they had a lot of questions about it.*”

(Daycare center 3)

The exploration of the green yards was a relational and situational process. One of the teachers illustrated this by telling us that after the green yards were installed, the children’s questions emerged so fast that she was happy that they had iPads and could search for information to answer the questions. Hence, the dynamic and situational engagement encouraged both children and adults to explore further and receive stimulus from the yard and the internet. In this way, the use of technology became intertwined with the use of the environment; it was not only seen as an activity that contradicts being outdoors. In addition to searching for information on portable computers, the daycare employees instructed children to bring insects inside in pots and examine them up close.

Loose materials, such as sticks, cones, and peat blocks, also enabled learning pre-academic skills, for instance, counting or recognizing different shapes or letters. The greening of the yard helped make these activities originate from the children themselves and from their own experiences, their own life-worlds [52]. The children could identify details in the yards, and the teachers and child nurses could use these as a starting point for learning and teaching situations.

“*You could find cones and sticks there [forest floor mat], and for some reason these sticks are absolutely fantastic when you have a lot of them. Our old trees drop a lot of sticks during storms. And then we just said, for instance, ‘look for three sticks’, and then they were searching for various colours or shapes in pairs, ‘look for three of this and four of that’. So we could take a lot of content from this for activities. For learning mathematics and language…*”

(Daycare center 1)

A few daycare employees pointed out that for some children, the trips to forests organized in the daycare center was their only everyday connection to the natural environment, as many came from families that mainly spent time in the city environment and shopping centers. Especially for these children, the greening of the daycare yard opened up new possibilities for everyday exploration—and the foundation for an environmental relationship and responsibility [30].

## 4. Discussion

### 4.1. Multiple Affordances of Green Yards

In this study, we indicated how green yards with diverse vegetation and high microbial load encourages children’s engagement with their everyday life-worlds in urban daycare centers. Affordances provide opportunities for not only functional actions, but also emotional experiences and psychological behaviors that can improve mental health and well-being [64]. First, we identified the various functional opportunities that the greening of the yards afforded the children; then we presented the dynamic aspects related to the greening from three main perspectives: Embodied experiences, involvement and exploration. Based on our results, the dynamic and emotional ways of engaging with nature enhance children’s well-being in daycare centers. Well-being is often derived through pleasant sensations and embodied experiences, such as breathing in the fresh air, smelling, or feeling nature [65]. Everyday experiences of engaging with nature situated in a daycare yard can open up possibilities not only for the well-being of the children, but also for the well-being of their environment. This well-being derives from personal involvement and emotional engagement in caring for our life-worlds [52,63]. Hence, environmental relationships appear to be constructed through diverse everyday dynamics: Through involvement, excitement, fascination—and becoming skilled in acting and being in the natural environment.

It has been suggested that positive, restorative experiences in the natural environment evoke a will to protect nature and promote environmental responsibility [30]. Here, the development of the environmental relationship is seen to be based on both the embodied and collective experiences in green yards and cognitive learning and exploration. Connectedness and involvement are often constructed through autotelic practices—practices that seem to have no goal or point, such as collecting cones or branches, organizing stones in different formations, or just lying down [52]. Intense use of green settings is considered to make places emotionally charged for children; our results confirmed that activities, such as planting, are hands-on activities that children value as meaningful. Greenery is associated with place attachment, which makes children more prepared to take on a long-lasting responsibility for their environment [31]. Just as Laaksoharju and Rappe [49] highlighted the role of trees as affordances for connectedness to place, green daycare or schoolyards may enable connectedness to place for children—and finally connectedness to nature.

Our study indicated that the green yard turned out to be a new useful working tool for teachers and child nurses. They used natural materials in various activities which they linked to the wider pedagogic goals of early childhood education. Green daycare or school grounds can be used as sites for outdoor learning as they provide a setting for hands-on, experimental learning [34]. While planters mostly enable teacher-led activities, forest floor vegetation, sod, and peat blocks also give inspiration for children’s self-guided and spontaneous play. The role of free play is emphasized as a key to a more bodily, emotional and sensuous interaction with nature [47,53]. Our study showed the importance of the microbially diverse, loose and modifiable elements that children could explore and manipulate in the yard. Peat blocks, sticks and cones were used in imaginative ways in constructive and symbolic play [26,31]. When planting vegetables or flowers, the children were practically involved with their everyday environment—their hands in the dirt, hosting billions of bacteria per gram. These results draw upon Nicholson’s [66] theory of loose parts which stresses that including variables or loose parts in the environment offers many opportunities for play and stimulates creativity that is unlikely to be found in settings with fixed elements. Children’s opportunities to manipulate their environment and build things provide several benefits, such as enhanced environmental learning and sense of place [24].

### 4.2. Implications

The importance of daycare and schoolyards for children’s unorganized “free” play and contact with the green environment is highlighted in urbanized societies, where children’s nature experiences are to an increasing degree organized by adults [2,53]. For instance, the findings of Zamani [25,26] suggest incorporating natural elements in playgrounds, as natural zones offered the most diverse spectrum of cognitive play and were supportive of different learning styles compared to the manufactured zone which offered the most functional and non-play behaviors. Based on our findings, such a spectrum of play with natural elements is also likely to increase the level of interaction with environmental microbiota. It is recommended that biodiverse, low-maintenance spaces are designed, which invite children to use green spaces according to their needs and allow child-directed, place-based playtime [49]. According to Fjørtoft and Sageie [38], the diversity of vegetation and topography is an important criterion in the planning and management of playscapes for children as it corresponded to function-related structures that afforded versatile play in natural playscapes.

Microbial diversity should also be taken into account when designing playgrounds as exposure to diverse environmental microbial communities has been shown to diversify the commensal microbiota and improve immunoregulation [45]. Our study indicated that it is possible to design green yards in a way that increases the diversity and abundance of safe health-associated environmental microbiota. All practical involvement, direct contact with natural materials enhances children’s exposure to environmental microbiota; a previous study in Finland [67] found higher skin microbiota diversity and the most dissimilar microbiota community composition in the children who attended nature-oriented daycare center compared to children in city-center or conventional daycare centers. Our study showed that green, biodiverse materials inspire children to play with, explore and manipulate them. If the novelty starts to wear off, teachers and child nurses may feed children’s imagination by giving them ideas and additional materials for play.

Current safety regulations substantially influence the design of daycare and school grounds. Meanwhile, playgrounds may have become less challenging and interesting for children [68]. The lack of empirical research on injuries has been seen as a major barrier to promoting change in playgrounds’ safety regulations [21]. In Finland, European Standards define specific safety requirements for playground equipment and protective surfacing that is meant for covering the base of playground equipment to reduce any impact from falls. In some of the daycare centers in this study, the green ground—forest floor mat or sod—was considered safer than the normally used ‘safe gravel’, which may easily cause wounds when children fall on it. The feeling of safety attached to natural elements was also illustrated by some children’s increased courage to climb or swing above the green ground. Through engagement with green yards, children develop practical skills of acting and being in natural environments, and this may further help make trips outside the classroom safer [47].

Bell and Dyment [20] conclude that school grounds affect all dimensions of children’s health, and if they are thoughtfully designed, they can become an essential factor in promoting health. Daycare yards and playgrounds within residential areas are even more important for children’s healthy everyday habits, since smaller children’s opportunities to spend time in the natural environment are dependent on their families. Recent guidelines to create and maintain natural areas for children [69] should be complemented by taking into account the environment’s microbiota-human commensal microbiota and health nexus [70]. In practice, the decision-making processes related to designing playgrounds are often driven by external constraints, such as budgets, licensing and space limitations, rather than children’s educational or health requirements [21]. To promote the greening of playgrounds, cities should see the higher installation and maintenance costs as investments in children’s healthy development rather than as extra costs. Our study indicated that the greening of daycare yards could be realized with reasonable effort and expense. Thus, there is a potential for widespread dissemination of the results. Informing planners, decision-makers, officials, and employees of the benefits of green cover are needed to enhance the willingness to design green yards and protect any existing vegetation in the building phase. Since forest floor vegetation is a slowly-renewable natural resource which erodes in highly used playgrounds, other microbially diverse landscaping materials are currently in development [44,45].

## 5. Conclusions

By combining the concept of affordance with measurements of microbial diversity in a novel way, we identified both the functional opportunities provided by the green daycare yards and the different dynamic aspects related to the greening. This study conducted in 13 groups in six urban daycare centers in Finland showed that the green yards inspired 3–5 years-old children’s play, increased their physical activity, and exposed children to high environmental biodiversity. The forest floor mat, sod, peat blocks, and planters offered embodied experiences of nature and enabled children’s multi-sensory exploration and diverse learning situations. The green materials encouraged both children’s spontaneous play and teacher-led activities linked to wider pedagogic goals. The dynamic and emotional ways of engaging with the natural environment enhanced the children’s well-being in the daycare centers—illustrated by positive impacts on mood, energy and motivation. The practical and emotional involvement with the green yards enabled the children to become skillful in using the natural environment as a play environment, in both imaginary play and physical activities. Furthermore, activities related to caring for the green yards and exploring them enhanced the development of environmental relationships and may lead to more responsible attitudes towards the natural environment.

The study results can be used in urban planning for designing biodiverse, health-enhancing yards at daycare centers and schools and other playgrounds. Compared to the previous greening projects, this study highlighted the importance of taking into account microbial diversity when designing playgrounds. Practical involvement with natural materials enhances children’s exposure to diverse environmental microbiota, which is associated with benefits to the immune system and health.

The limitations of this study are related to the collection of only survey and interview data based on daycare employees’ and parents’ observations and perceptions of the greening. In the future, more participatory research based on direct observation methods is needed in order to analyze in detail how children use green yards and what kind of affordances natural materials enable. The dynamic engagement should be studied not only from the perspective of children and green materials, but also from that of the personnel and children acting together in the green yards, for instance, how changes in children’s and adults’ moods are connected to each other, and how green yards also motivate adults and affect their practices. The qualitative analysis could be mixed with quantitative methods, such as using accelerometers or video observations to analyze the children’s activities in the green yards and comparing them to those in traditional yards [27]. We also encourage further studies to confirm the relationships between environmental microbiota, human commensal microbiota, and physical and mental health.

## Figures and Tables

**Table 1:** Permutational Multivariate Analysis of Variance showed that the intervention changed the community composition of bacteria in the daycare yard at the OTU level and that the bacterial communities in the material types differed (mineral soil, sod, forest floor).

	Df	Sums of Sqs	Mean Sqs	F. Model	R^2^	*p* Value
intervention	1	1.73470000	1.73468	7.74250000	0.30811	0.001
material type	1	0.9829	0.98286	4.3868000	0.17457	0.001
residuals	13	2.912600000000	0.22405	0.51732		
total	15	5.6302000	1.00000			

OTU: operational taxonomic unit with a 97% identity; Df: degrees of freedom; Sqs: sum of squares.

**Table 2:** Number of the survey respondents and interviewees in the study.

	Survey	Interview
Daycare personnel	13	12
Parents	49	0

**Table 3:** Functional affordances enabled by green daycare yards.

Environmental Qualities that Support Affordance	Affordance
	**Physical activity**
Sod, forest floor mat afforded	rolling
	creeping, crawling
	doing somersaults, cartwheels, other physical movements
	running, jumping
	playing ball games/other active games
	climbing, swinging ^1^
Peat blocks afforded	jumping down/over
	walking on
	carrying, throwing
	**Multi-sensory experiences**
Sod, forest floor mat, peat blocks, planters afforded	touching, plucking
	smelling
	sensory exercises
Sod, forest floor mat, peat blocks afforded	lying, sitting
Planters, forest floor mat afforded	tasting
Forest floor mat afforded	making sounds (sticks, cones), listening
	**Diverse play**
Sod, forest floor mat afforded	playing with toys (e.g., animals, cars)
	pretend play (e.g., playing house, playing animals)
	playing Kim’s game (memory game)
Peat blocks afforded	building (e.g., walls, forts, huts)
	**Art and crafts**
Forest floor mat, sod, planters afforded	doing art and crafts
	**Nature exploration**
Forest floor mat, sod, planters afforded	searching for bugs, worms, snails, etc.
	examining (using a magnifying glass)
	observing, wondering
	identifying species
	learning concepts related to nature
Planters afforded	planting, taking care of plants
	**Pre-academic skills**
Forest floor mat, peat blocks afforded	learning pre-math skills
	learning pre-reading skills

^1^ soft ground.
